# Levofloxacin is phytotoxic and modifies the protein profile of lupin seedlings

**DOI:** 10.1007/s11356-017-9845-0

**Published:** 2017-08-10

**Authors:** Aleksandra Orzoł, Agnieszka I. Piotrowicz-Cieślak

**Affiliations:** Department of Plant Physiology, Genetics and Biotechnology, Faculty of Biology and Biotechnology, University of Warmia and Mazury in Olsztyn, Oczapowskiego 1A, 10-718 Olsztyn, Poland

**Keywords:** Environmental exposure, Enzyme activity, Levofloxacin, Lupin seedlings, Protein profile, Reactive oxygen species

## Abstract

The toxicity of levofloxacin to yellow lupin plants was evaluated in this study. Recommended indexes of plant (roots and shoots) growth were determined and new indexes were proposed which better characterise the phytotoxicity of levofloxacin. These were, in particular, the activity of antioxidative enzymes, the content of free radicals, as well as the root protein content and the root protein profile. The results showed that levofloxacin considerably affected EC_50_, measured as the activity of catalase in roots, and leaves (1.05 and 0.069 mM, respectively). The activity of peroxidase in the roots and the dry weight of seedlings were the least sensitive parameters (EC_50_ was 1.8 and 1.76 mM, respectively). Units of toxicity clearly showed that the activity of catalase is a better measure of toxicity for low concentrations of the drug, and it is a better index of plant physiological state than the morphological parameters of seedlings. Moreover, levofloxacin changed the location of free radicals and the protein profile in plants. The changes in location of reactive oxygen species in roots were an important symptom of the drug toxicity to lupin seedlings. Our results have shown that the toxicity of levofloxacin was manifested mainly by changes in the protein profile. The content of the glyceraldehyde-3-phosphate dehydrogenase, 14-3-3-like protein A, expansin-B3-like precursor, fructose-bisphosphate aldolase, lipoxygenase, nucleotide-binding subunit of vacuolar ATPase and pyruvate dehydrogenase were found to decrease. On the other hand, plant exposure to levofloxacin resulted in an increase in the content of enolase, protein LlR18A, class III chitinase, ascorbate peroxidase, aspartate aminotransferase, alcohol dehydrogenase 1, leghemoglobin reductase-like 17 and heat shock cognate protein 80-like.

## Introduction

Excessive use of antibiotics in general and in veterinary medicine has resulted in the contamination of the natural environment with these substances (EEA 2010). Of all the veterinary antibiotics (VAs), only 20% are used to treat infections; the other 80% are used to prevent diseases or to promote animal growth (US FDA 2015). The dosage of VAs varies (from 3 to 220 g kg^−1^) depending on animal size and the type of antibiotic used (Health Canada 2002). After being administered, VAs are largely (from 10 to 90%) eliminated from the body to the environment either in an unchanged form or as active metabolites (Elmund et al. 1971; Halling-Sørensen et al. 2001). Antibiotics have been found in animal faeces and in soil in concentrations ranging from 0.01 to 1420 mg kg^−1^ (Pan and Chu 2016). In aquaculture, pharmaceuticals are added to water in fish ponds, and their concentrations in water and lake deposits are high (Li et al. 2012). An increase in the concentration of VAs and their metabolites in the environment can have a negative impact on land organisms (Pan and Chu 2016). The most common antibiotics used in veterinary medicine include tetracyclines, fluoroquinolones, macrolides and sulphonamides (EMA 2015).

Fluoroquinolones (FQs) are a new, synthetic group of antibiotics with strong antibacterial action and a broad spectrum of anti-pathogenic activity. FQs are used for the treatment of infections of the urinary tract, the gastrointestinal system, the respiratory tract and the skin in medicine (EMA 2017) and in intensive breeding of cattle, poultry, pigs, fish, as well as dogs and cats (EMEA 2007). Fluoroquinolones are highly stable, due to which they are not wholly metabolised in the body. Their residues can be eliminated, and they can find their way to wastewater treatment plants or to the environment (Watkinson et al. 2007). The toxicity of FQs towards microorganisms limits the processes of antibiotic biodegradation in water and in soil (Robinson et al. 2005). They have been found in crude wastewater (2573 ng L^−1^), secondary wastewater (1013 ng L^−1^) and in sludge (18.4 mg kg^−1^) (Jia et al. 2012). Manure used as fertiliser (faeces of chickens, pigs and cows) is the main source of contamination of soil with antibiotics (Li et al. 2013). Di- and trivalent cations (Ca^2+^, Mg^2+^ and Al^3+^) form stable complexes with fluoroquinolones. The chelates are strongly adsorbed in soil, and they cannot be leached out of it (Turel et al. 1996; Djurdjević et al. 2007). The concentration of FQs in the soil fertilised with manure ranges from 0.01 to 0.4 mg kg^−1^ of soil, and it remains at this level for several months (Karcı and Balcıoğlu 2009). Boxall et al. (2006) proved the stability of enrofloxacin in soil; its half-life was longer than 152 days. Furthermore, Golet et al. (2002) applied ciprofloxacin and norloxacin to soil at a concentration ranging from 0.29 to 0.40 mg kg^−1^; after nearly 2 years in the soil, there was still 93 and 75% of the drugs, respectively.

Fluoroquinolones are divided into four groups (generations) in which antibiotics differ by the structure and the spectrum of action (Soni 2012). Third-generation FQs include the l-isomer of ofloxacin—levofloxacin (LEV). LEV is characterised by good infiltration of target tissues and is used to control intracellular pathogens. The drug is eliminated from the body through the kidneys, usually as the active antibiotic. LEV is metabolised to a small extent and its inactive metabolites, i.e. demethyllevofloxacin and levofloxacin *N*-oxide, account only for < 5% of the dose applied (Levaquin® 2008). After being eliminated, the antibiotic is introduced to soil, surface and ground waters and to lake deposits (Tonga et al. 2009). Boxall et al. (2006) proved that antibiotics are absorbed from soil by roots of carrot and lettuce. The consequences of the presence of fluoroquinolones in the environment have not been fully revealed, but they are known to be toxic to plants (Frade et al. 2014).

Since levofloxacin effectively enters inside the cell and is used to control intracellular pathogens, changes caused by its toxic action will become apparent at the cellular level earlier than anywhere else. When evaluating the environmental risk caused by xenobiotics, the majority of studies have focused on toxic chemicals. However, biochemical changes in plants can be detected earlier than injuries, or the considerable decrease in plant growth and viability. Therefore, antibiotic toxicity to lupin was evaluated in this study with the use of standard endpoints recommended in guidelines, which include biomass parameters. Lupin is a common fodder crop (Lefroy and Rydberg 2003), and biochemical contaminants accumulated in it can be transferred to the food chains comprising farm animals. Moreover, it was recently proposed as a component of functional foods and a health-promoting plant (Pilvi et al. 2006). Considering this, it is important to understand the reactions of this plant to environmental pollutants. Furthermore, new endpoints were calculated in this study which had not been examined before, namely, oxidative stress enzymes (guaiacol peroxidase, catalase), distribution of reactive oxygen species (ROS) in cytoplasm and protein contents and profiles.

## Material and methods

### Germination and growth of seedlings

Seeds of yellow lupin (*Lupinus luteus* L.), Dukat cultivar, were used in the study. The seeds germinated for 7, 9 and 12 days on PHYTOTOXKIT test plates (MicroBioTests Inc., Mariakerke (Gent), Belgium). Before germination, the seeds were surface-sterilised by immersing them in 1% sodium hypochlorite for 2 min; the seeds were then washed in distilled water for 3–4 min. Next, 90 mL of soil was added to the plates and they were then watered with 27 mL of water (control) or 27 mL of levofloxacin solutions at different concentrations (Sigma-Aldrich): 0.01, 0.05, 0.1, 0.5, 1, 1.5, 2 and 2.5 mM (i.e. 0.7, 3.5, 7, 35, 70, 105, 140, 175 mg kg^−1^ of soil). The soil was covered with Whatman no. 1 filter paper on which seeds were placed. The biotest was carried out for 7, 9 and 12 days at room temperature. Roots and shoots of the seedlings were measured with the ImageJ tool software. Fresh and dry weights were determined.

### Activity of guaiacol peroxidase

Plant extracts were prepared on ice. Roots, shoots and leaves (500 mg) of the seedlings which had grown for 7, 9 and 12 days in soil with an addition of water or levofloxacin were homogenised for 30 min in a buffer solution (0.1 M Tris-HCl, Sigma-Aldrich; 8.75% polivinylpyrrolidone, Sigma-Aldrich; 0.1 M KCl, 0.28% Triton X-100, Sigma-Aldrich). The samples were centrifuged for 30 min at 4000×*g* at 4 °C. The supernatant was purified with membrane syringe filters with a pore diameter of 0.45 μm. Protein in the samples was assayed by the Lowry method (Lowry et al. 1951). The activity of peroxidase was determined spectrophotometrically (CECLI, CE2021 2000 Series) in the reaction mixture containing 100 μL of 1% guaiacol (Sigma-Aldrich), 2 mL 0.1 M KH_2_PO_4_ (Chempur), 50 μL of the supernatant and 20 μL 0.06% H_2_O_2_ (Chempur). The absorbance growth rate was measured at room temperature at the wavelength of 470 nm. One unit corresponds to oxidation of 1 μM H_2_O_2_ for 1 min.

### Activity of catalase

Plant extracts were prepared on ice. Roots, shoots and leaves (500 mg) of the seedlings which had grown for 7, 9 and 12 days in soil with an addition of water or levofloxacin were homogenised in phosphate buffer solution pH 7, which contained 10 g L^−1^ polivinylpyrrolidone (PVP, Sigma-Aldrich), 0.2 mM EDTA (Sigma-Aldrich) and 10 mL L^−1^ Triton X-100 (Sigma-Aldrich). The samples were centrifuged for 20 min at 12000×*g* at 4 °C. The supernatant was then carefully separated from the sediment. Protein in the samples was assayed by Lowry’s method (Lowry et al. 1951). The activity of catalase was determined spectrophotometrically (CECLI, CE2021 2000 Series) with a reaction mixture containing 50 mM phosphate buffer, pH 7 and 15 mM H_2_O_2_. The absorbance was measured for 10 min at room temperature at the wavelength of 240 nm. One unit corresponds to oxidation of 1 μM H_2_O_2_ for 1 min.

### Reactive oxygen species

Seeds of yellow lupin were germinated in soil watered with deionised water. After 7 days, the seedlings were transferred to new, sterile PHYTOTOXKIT test plates filled with control soil and with a 2.5 mM solution of levofloxacin. After 24 and 48 h, roots of the seedlings were incubated in the dark for 30 min in 0.1 M PBS at pH 7.4 (phosphate-buffered saline), with 10 μM 2′,7′- dichlorodihydrofluorescein diacetate (H_2_DCF-DA). Subsequently, they were washed several times with a 0.1 M PBS solution at pH 7.4. 2′,7′-Dichlorodihydrofluorescein diacetate is a lipophilic and non-fluorescent compound which freely infiltrates cell interiors. H_2_DCF-DA is degraded in live cells by intracellular esterases to lipophobic dichlorofluorescein (DCF), which emits a fluorescent signal. DCF reacts with reactive oxygen species (ROS), as well as with reactive nitrogen species and with lipid peroxides. The fluorescence of DCF was measured with a Leica TCS SP5 confocal scanning laser microscope with LAS software (Leica Application Suite 2.0.2 build 2038). Oxidised dichlorofluorescein fluoresces at the absorption maximum of 525 nm.

### Isolation of protein and 2D electrophoresis-PAGE

Plant extracts were prepared on ice. The roots of yellow lupin seedlings (300 mg) grown for 12 days in soil with an addition of water and 2.5 mM of solution of levofloxacin were homogenised in a buffer which contained 7.4 M of urea, 2.1 M of thiourea, 62 mM of CHAPS (Sigma-Aldrich), 8 mM of DTT (Sigma-Aldrich), 0.24% of Triton X-100 (Sigma-Aldrich), 16.5 mM of Trizma base (Sigma-Aldrich), 21 mM of Trizma hydrochloride (Sigma-Aldrich), 1% of ampholytes pH 3–10 (Bio-Rad) and a cocktail of proteases (Sigma-Aldrich) (Badowiec et al. 2013). The samples were shaken for 45 min and subsequently centrifuged for 10 min at 18000×*g* at 4 °C. The supernatant was frozen at −80 °C and then lyophilised. The lyophilisate was purified with ReadyPrep™ 2-D Cleanup Kit (Bio-Rad). The isolated proteins were dissolved in a buffer for rehydration which contained 7 M of urea, 2 M of thiourea, 2% of CHAPS, 0.5% of ampholytes pH 3–10, 80 mM DTT and 0.002% of bromophenol blue. Two-way electrophoresis was carried out in two stages. First, rehydration and isoelectric focusing was carried out with a PROTEAN IEF Cell (Bio-Rad). A protein solution (70 μg of protein) was added to a well in the focusing plate, IPG strips (ReadyStrip™ 7 cm, pH 3–10; Bio-Rad) were placed and a 12-h active rehydration was carried out. After rehydration was completed, isoelectric focusing was carried out (conditions: preparatory stage S1—250 V / 15 min; voltage increase stage S2—4000 V / 2 h 30 min; isoelectric focusing S3—rapidly increasing voltage up to 20,000 V). After isoelectric focusing was completed, IPG strips were equalised in buffer I (6 M urea, 2% SDS, 0.375 M Tris-HCl with pH 8.8, 20% glycerol, 130 mM DTT; Bio-Rad) and buffer II (6 M urea, 2% SDS, 0,375 M Tris-HCl with pH 8.8, 20% glycerol, 135 mM iodoacetamide; Bio-Rad) for 10 min.

The second stage involved separation of proteins in 10% polyacrylamide gels (7.0 cm × 10.0 cm) in an electrophoresis apparatus (Mini PROTEAN Tetra System; Bio-Rad). Electrophoresis was carried out for 1 h, with 45 min in alternating current: 90 V (15 min) and 120 V. After the electrophoresis was completed, gels were dyed in colloidal solution of Comassie Brilliant Blue G-250 (Sigma-Aldrich). The gels were visualised with a Gel Doc EZ Imager scanner (Bio-Rad). The protein maps were analysed in the PDQuest™ Basic program (2-D Gel Analysis Software; Bio-Rad).

### Digesting proteins and LC-MS-MS/MS analysis

Spots with proteins were carefully cut out from the polyacrylamide gels. After reduction and blocking the reduced bridges, a mixture of peptides was obtained with the use of trypsin. The mixture was separated by liquid chromatography (LC), and the mass of peptides and their fragments was determined in a mass spectrometer (Orbitrap LC-MS-MS/MS; Thermo). The NCBI and UnitProt databases were searched for MS/MS spectra. The proteins were identified based on PMF (peptide mass fingerprint) with the MASCOT search engine.

### Statistical analysis

One-way analysis of variance (ANOVA) followed by Tukey’s comparison post hoc test (*p* ≤ 0.01) was applied to evaluate differences between controls and treatments. Effective concentrations (EC_*x*_) calculations (Alexander et al. 1999) were carried out for inhibition of shoot and root growth, dry and fresh weight and enzyme activity, and the results were expressed in mM. EC_10_, EC_25_, EC_50_ and EC_90_ express concentrations of the antibiotic resulting in 10, 25, 50 and up to 90% of the toxic effect. The effective concentration (EC) was computed from the following equation:

$$ {\mathrm{EC}}_x\kern0.5em =\kern0.5em C\kern0.5em +\kern0.5em \frac{\left(A\kern0.5em -\kern0.5em 50\%\max \kern0.5em \mathrm{response}\right)\kern0.5em \times \kern0.5em X}{y} $$

where *x* = *D*_(max concentration)_, *C*_(min concentration)_ = difference of concentration, *y* = *A*_(max response)_, *B*_(min response)_ = difference of response. The EC values expressed in μM were transformed to the measured toxicity units (TU): TU_10, 25, 50, 90_ = (1/EC_10, 25, 50, 90_) × 100.

## Results

**Fig. 1:**
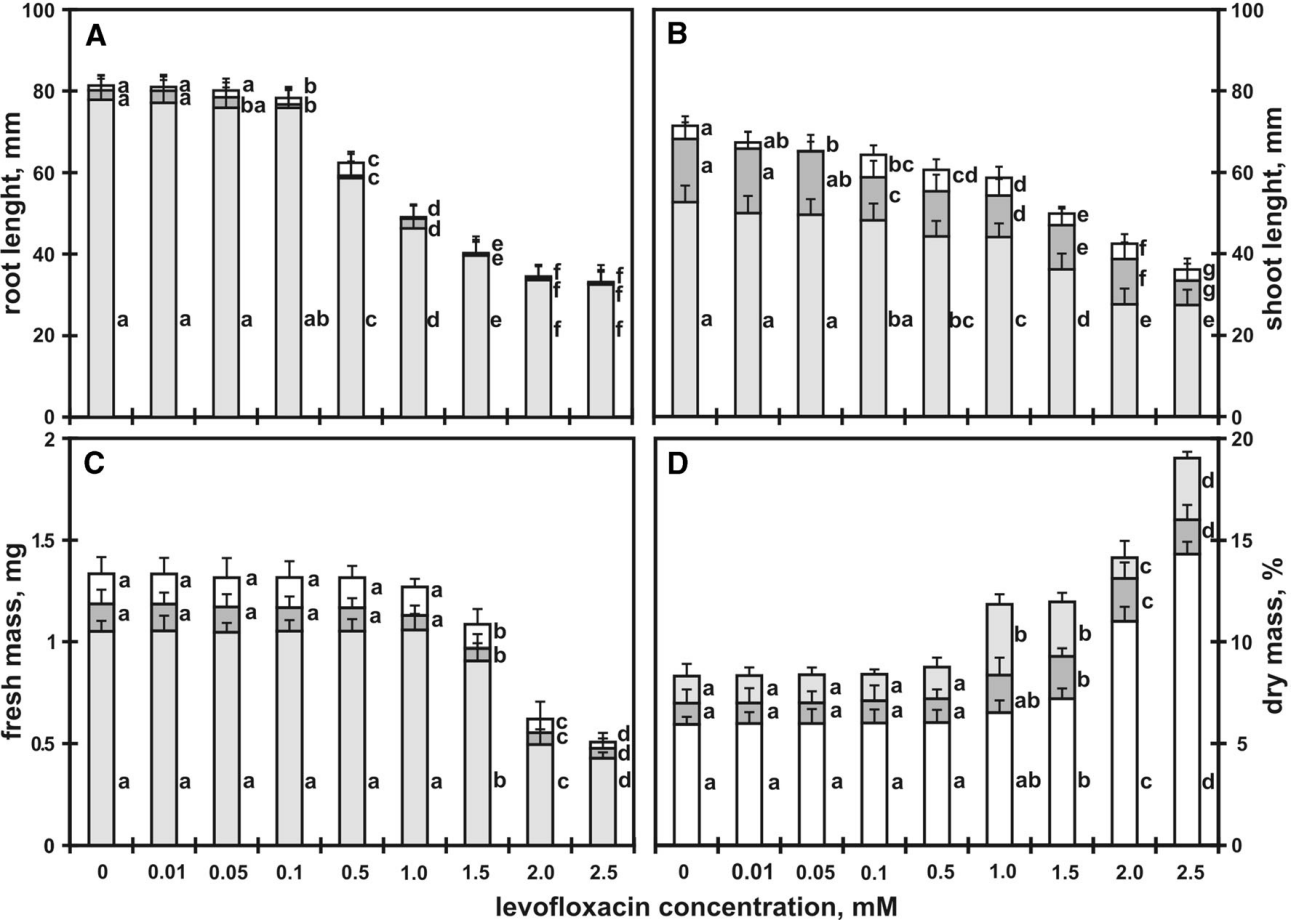
Roots (**a**) and shoots length (mm) (**b**), fresh (mg) (**c**) and dry mass (%) (**d**) of *Lupinus luteus* L. after 7 (■), 9 (■) and 12 (□) days on soil supplemented with different levofloxacin concentrations (**c** control; 0.01, 0.05, 0.1, 0.5, 1, 1.5, 2, 2.5 mM). Means with the same letter are not significantly different from each other (Tukey’s test, *p* ≤ 0.01)

The toxicity of levofloxacin (LEV) towards seedlings of yellow lupin (*L. luteus* L. cv. Dukat) was examined. The seedlings grew for 7, 9 and 12 days on control soil (water) and on soil contaminated with LEV at various concentrations (0.01, 0.05, 0.1, 0.5, 1, 1.5, 2, 2.5 mM, i.e. 0.7, 3.5, 7, 35, 70, 105, 140, 175 mg kg^−1^ of soil). The following morphological parameters of the seedlings were evaluated: length of roots and shoots, seedling fresh and dry weight (Fig. 1) and the physiological and biochemical parameters, including activity of enzymes (catalase and peroxidase), protein profile and location of free radicals.

### Morphological parameters

Lupin seed germination, manifested by protrusion of the radicle through the seed coat, occurred at 1–1.5 days after sowing. The longest roots were observed in seedlings which had grown for 7, 9 and 12 days on control soil; they were 78, 80 and 81 mm long (Fig. 1a), respectively. The roots of 7-, 9- and 12-day-old seedlings which had grown in soil with 0.1 mM of solution of levofloxacin were on average slightly shorter than the roots of the control seedlings by 3, 4 and 4%, respectively. With the highest LEV concentration applied (2.5 mM), inhibition of root growth was much stronger—58, 59 and 60% compared to the roots of the control seedlings in 7-, 9- and 12-day-old seedlings, respectively (Fig. 1a).

The response of shoots to LEV was comparable to the reaction of roots. In control seedlings grown for 7, 9 and 12 days, shoot lengths were 53, 68, 71 mm long, respectively (Fig. 1b). The smallest concentration of LEV (0.01 mM) reduced the shoot length in 12-day-old seedlings by 5% (Fig. 1b), and the highest LEV concentration (2.5 mM) resulted in a 49% reduction of shoot length of the 12-day-old seedlings. The EC_50_ indexes for the root and shoot elongation were 0.7 and 1.3 mM (Table 1), respectively.

**Table 1:** Effect of levofloxacin on growth and enzyme activity parameters in 7, 9 and 12 days in yellow lupin assays

	EC_10_			EC_25_			EC_50_			EC_90_		
Length	Root	Shoot		Root	Shoot		Root	Shoot		Root	Shoot	
7 days	0.15	0.009		0.25	0.23		0.65	1.23		1.19	1.8	
9 days	0.12	0.007		0.2	0.1		0.65	1.23		1.7	2.15	
12 days	0.12	0.008		0.2	0.2		0.67	1.26		1.7	2.23	
Fresh weight	Seedlings											
7 days	1.24			1.5			1.68			2.0		
9 days	1.12			1.37			1.67			2.0		
12 days	1.03			1.38			1.69			2.3		
Dry weight	Seedlings											
7 days	0.65			0.7			1.78			2.37		
9 days	0.73			1.5			1.7			2.3		
12 days	1.18			1.6			1.8			2.3		
Catalase activity	Roots	Shoots	Leaves	Roots	Shoots	Leaves	Roots	Shoots	Leaves	Roots	Shoots	Leaves
7 days	0.13	0.54	0.002	0.25	0.68	0.005	1.52	0.8	0.069	2.3	2.26	1.9
9 days	0.15	0.58	0.003	0.27	0.7	0.083	1.15	0.83	0.1	2.35	2.25	2.2
12 days	0.11	0.56	0.002	0.2	0.69	0.007	0.49	0.86	0.2	2.32	2.23	2.31
Peroxidase activity	Roots	Shoots	Leaves	Roots	Shoots	Leaves	Roots	Shoots	Leaves	Roots	Shoots	Leaves
7 days	0.9	0.008	0.04	1.5	0.7	0.7	1.8	1.6	1.6	2.3	2	2
9 days	0.8	0.009	0.01	1.5	0.75	0.7	1.8	1.65	1.6	2.3	2.13	2.15
12 days	0.7	0.1	0.009	1.3	0.08	0.65	1.78	1.72	1.63	2.3	2.2	2.3

EC_10_, EC_25_, EC_50_ and EC_90_ values are expressed in mM

The fresh mass of seedlings decreased with an increasing concentration of levofloxacin. The weight of seedlings grown for 12 days in soil with 1.5 and 2.5 mM LEV solutions was smaller than the control seedlings by 18 and 62%, respectively (Fig. 1c).

The opposite pattern was observed in the case of seedling dry weight, i.e. it increased with increasing concentrations of LEV solutions (Fig. 1d). The dry mass of 12-day-old seedlings grown in soil contaminated with a 2.5 mM was greater than the control seedlings by 58%. Units of toxicity for dry and fresh weight of biomass ranged from 0.04 to 0.1, depending on LEV concentration and seedling age (Table 2).

**Table 2:** Toxicity units (TU_10, 25, 50, 90_ = (1/EC_10, 25, 50, 90_) × 100) calculated on the basis of levofloxacin concentration for 12 days

	TU_10_			TU_25_			TU_50_			TU_90_		
Length	Roots	Shoots		Roots	Shoots		Roots	Shoots		Roots	Shoots	
	0.77	12.5		0.46	0.57		0.15	0.08		0.07	0.05	
Fresh weight	Seedlings											
	0.09			0.07			0.06			0.05		
Dry weight	Seedlings											
	0.12			0.08			0.06			0.04		
Catalase activity	Roots	Shoots	Leaves	Roots	Shoots	Leaves	Roots	Shoots	Leaves	Roots	Shoots	Leaves
	0.77	0.18	43	0.42	0.14	3.2	0.09	0.12	0.81	0.04	0.04	0.05
Peroxidase activity	Roots	Shoots	Leaves	Roots	Shoots	Leaves	Roots	Shoots	Leaves	Roots	Shoots	Leaves
	0.13	2.6	5.1	0.07	0.20	0.15	0.06	0.06	0.06	0.04	0.05	0.05

The value EC_10, 25, 50, 90_ is expressed in μM

### Peroxidase and catalase activity

**Fig. 2:**
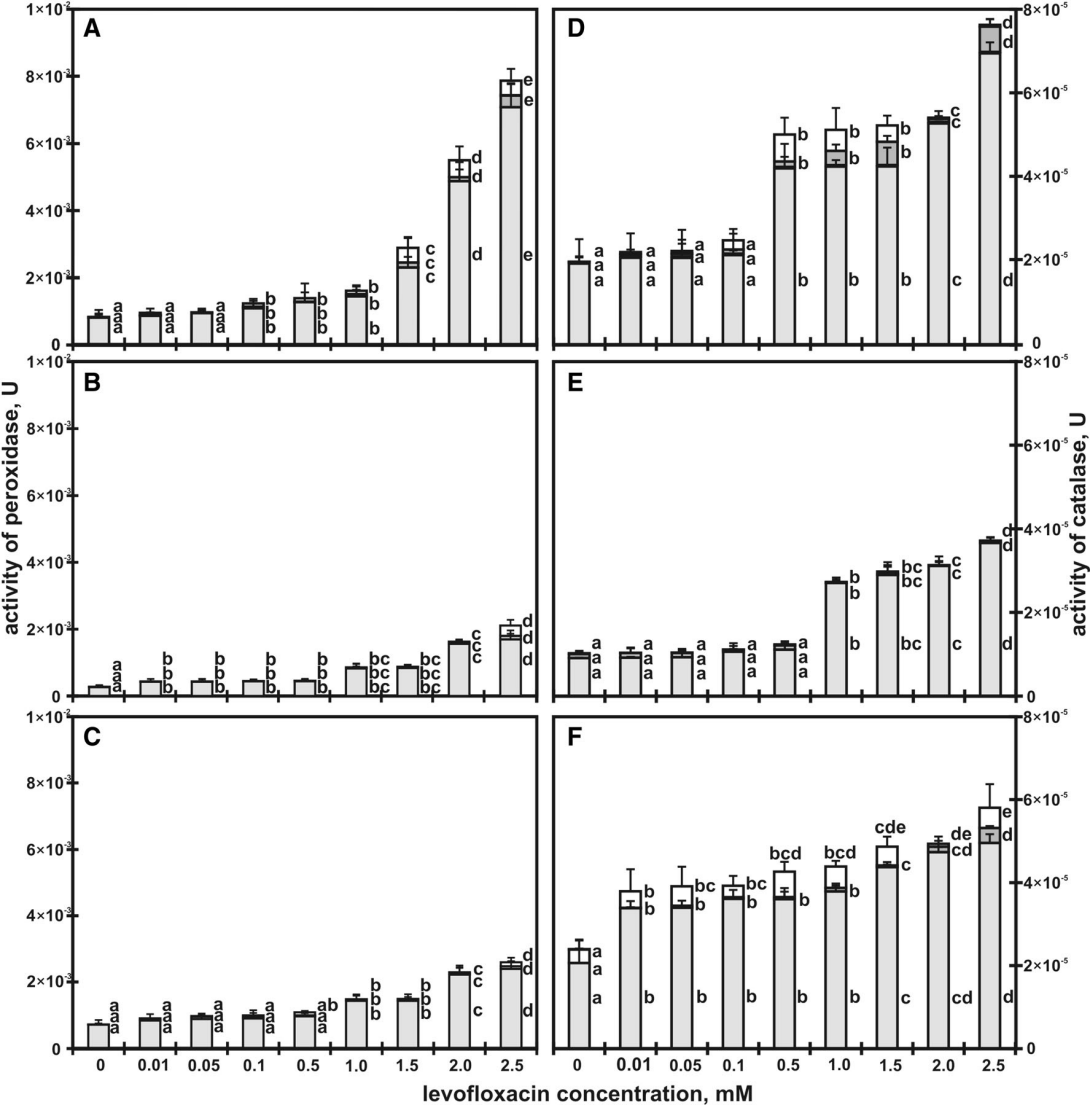
Activity of guaiacol peroxidase in roots (**a**), shoots (**b**) and leaves (**c**) and activity of catalase in roots (**d**), shoots (**e**) and leaves (**f**) (*U* one unit of enzyme activity corresponds to the oxidation of 1 mM H_2_O_2_ for 1 min) of *Lupinus luteus* L. after 7 (■), 9 (■) and 12 (□) days on soil supplemented with different levofloxacin concentrations (**c** control; 0.01, 0.05, 0.1, 0.5, 1, 1.5, 2.5 mM). Means with the same letter are not significantly different from each other (Tukey’s test, *p* ≤ 0.01)

The activity of guaiacol peroxidase (Fig. 2a–c) and catalase (Fig. 2d–f) in roots, leaves and shoots of seedlings grown in control soil (water) and in soil contaminated with levofloxacin.

The highest activity of peroxidase was recorded in roots of seedlings grown in soil with 2.5 mM of LEV (Fig. 2a). The activity of this enzyme in 7-, 9- and 12-day-old seedlings was nine times higher than in the control seedlings. The toxicity indexes (TU) 10 for the activity of the enzyme were very high: 2.6 and 5.1 for shoots and leaves, respectively (Table 2).

The enzyme activity in leaves and shoots of 12-day-old seedlings grown in soil with the lowest LEV concentration (0.01 mM) was 15 and 35% compared to the leaves and shoots of the control seedlings, respectively (Fig. 2b, c). The activity of peroxidase in leaves and shoots of the seedlings grown in soil with the highest concentration of LEV (2.5 mM) increased 4- and 8-fold, respectively. EC_50_ for the activity of peroxidase in roots, shoots and leaves of 12-day-old seedlings was 1.78, 1.72 and 1.63 mM, respectively (Table 1).

The catalase activity in 12-day-old lupin seedlings grew with increasing concentrations of LEV (Fig. 2d–f). In the case of leaves (but not roots and shoots), this rise was clearly visible even with the lowest LEV concentration. The largest increase in the enzyme activity (75, 72 and 73%) was observed in the shoots of 7-, 9- and 12-day-old seedlings grown in soil with an addition of 2.5 mM solution of LEV. EC_50_ determined for catalase was 1.1, 0.8 and 0.1 (Table 1) for roots, shoots and leaves, respectively. The toxicity index for catalase was highest for leaves (Table 2).

### Location of reactive oxygen species

**Fig. 3:**
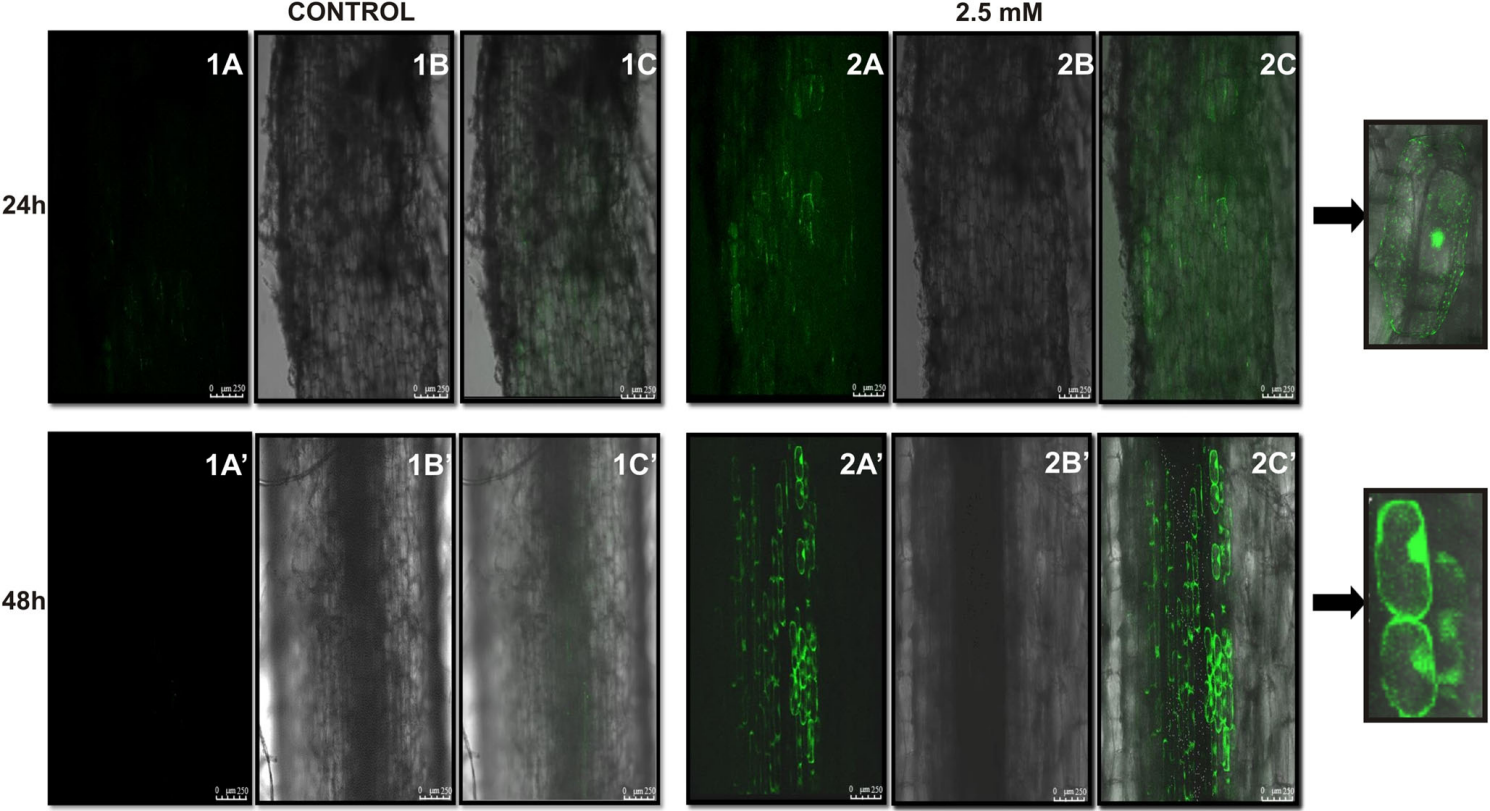
The control roots of 7-day-old (photo 1A, 1B and 1C) and 8-day-old (photo 1A’, 1B’ and 1C’) seedlings and the roots of 7-day-old seedlings that have grown by 24 h (photo 2A, 2B and 2C) and 48 h (photo 2A’, 2B’ and 2C’) in the soil with the addition of 2.5 mM concentration of levofloxacin. The results obtained were created by ROS location shooting performance at different depths preparation in the *Z* axis (Series Z) by way of a confocal microscope scan capture. *A*, *A’* the fluorescence image; *B*, *B’* without fluorescence image; *C*, *C’* overlapping images

The distribution of reactive oxygen species (ROS) in roots is presented in Fig. 3. The photographs were created by superimposing all photos taken at different depths of the preparation (snapshot). The reactive oxygen species in roots of 7-day-old seedlings which had grown for 24 h in soil contaminated with 2.5 mM LEV was seen to increase rapidly compared to roots of control seedlings (Fig. 3, 2A–2C). The intensity of fluorescence in the elongation zone was uniform in the material under study. At the beginning of the analyses period (24 h), ROS occurred within the whole interior of root cells. Extended exposure to the stressor (48 h) resulted in saturation of fluorescence in the roots under study (Fig. 3 2A’–2C’). Fluorescence in root cells was no more uniform, and ROS were found to accumulate along the cell wall. Fluorescence was also observed in the control roots, but its intensity was very low (Fig. 3 1A–1C, 1A’–C’).

### 2D-PAGE electrophoresis

**Fig. 4:**
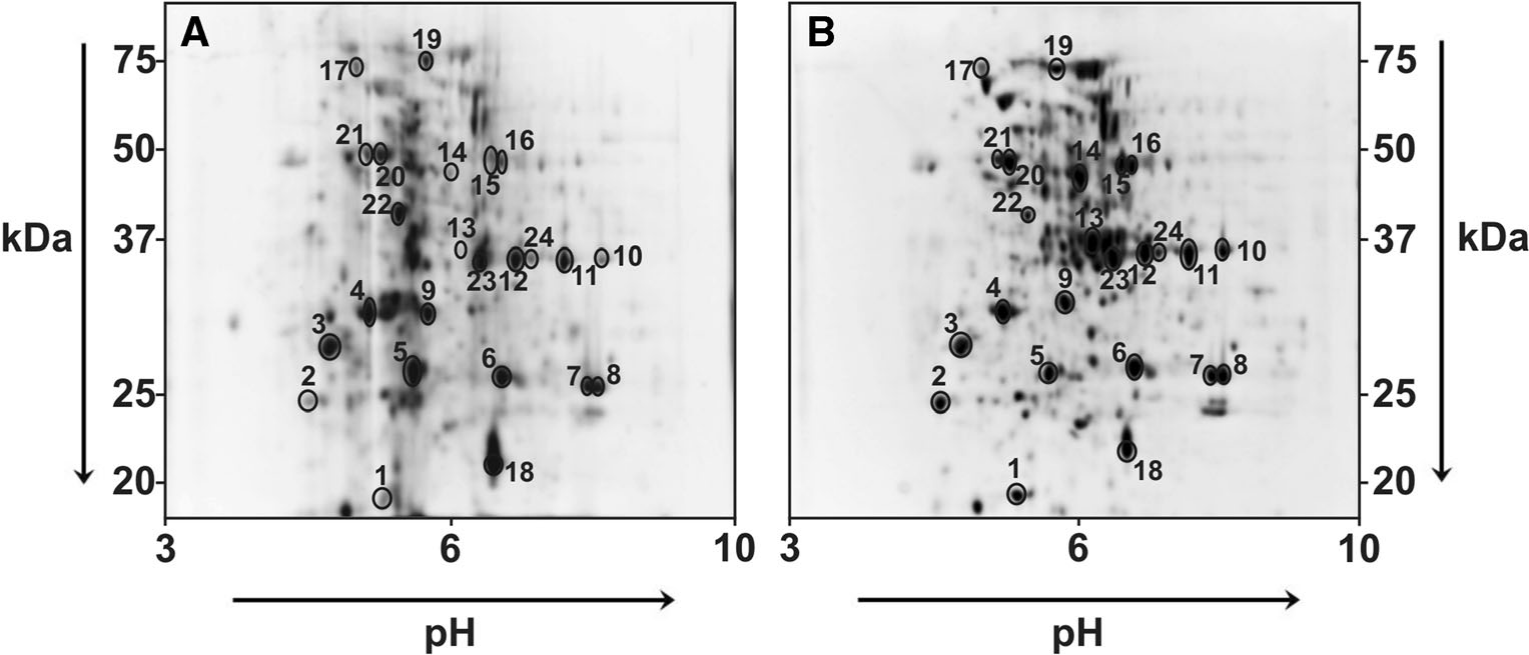
2D electrophoresis of proteins of root lupin (*Lupinus luteus* L.) without (**a**) and with (**b**) the levofloxacin at the concentration of 2.5 mM of soil. Protein separation was conducted at pH 3–10

**Fig. 5:**
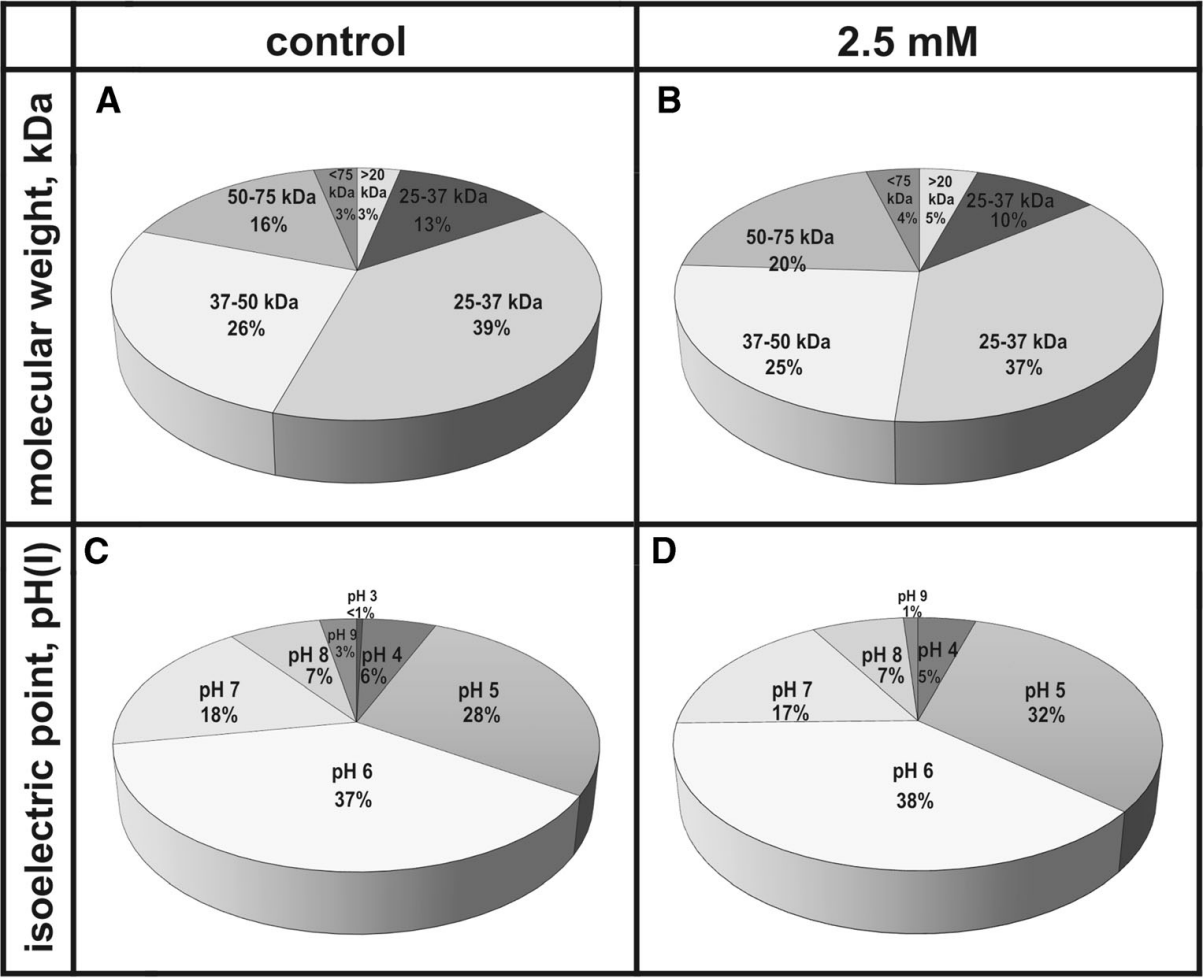
Pie chart showing the molecular mass distribution (kDa) of proteins from control roots (**a**) and the levofloxacin affected roots (**b**) at the concentration of 2.5 mM of soil and the pH range of proteins from control roots (**c**) and the levofloxacin-affected roots (**d**) at the concentration of 2.5 mM of soil

The effect of selected concentrations of levofloxacin on the yellow lupin proteome was analysed in this study. The 2D maps obtained which present the protein content in 12-day-old roots of seedlings which grew in control soil (water) and in soil with addition of 2.5 mM of LEV solution revealed differences in the amount and content of the isolated proteins (Fig. 4a, b). There were 205 and 261 proteins, respectively, found in gels made from control roots and from those growing in soil with an addition of levofloxacin. The maps created with control roots in the mass range from 25 to 37 kDa revealed the most abundant presence of proteins (79) of all the analysed samples (Fig. 5a). The number of proteins with molecular weights smaller than 20 kDa and larger than 75 kDa was the smallest. The number of proteins visualised in the control map at pH 6 was 75, which accounted for 35% of all the proteins obtained (Fig. 5b). The smallest number of proteins was found in the control gel in the area with pH 3. A 2D map made for roots of seedlings grown in soil contaminated with 2.5 mM LEV showed that proteins with a molecular weight between 25 and 37 kDa accounted for 37% of all proteins (Fig. 5c). Proteins with a molecular weight above 75 kDa accounted for only 4% of all proteins. The largest number of proteins (100) in the analysed gel was found at pH 6 (Fig. 5d). Three proteins (1% of all proteins obtained) were visualised at pH 9.

### Identification of antibiotic-responsive proteins

**Fig. 6:**
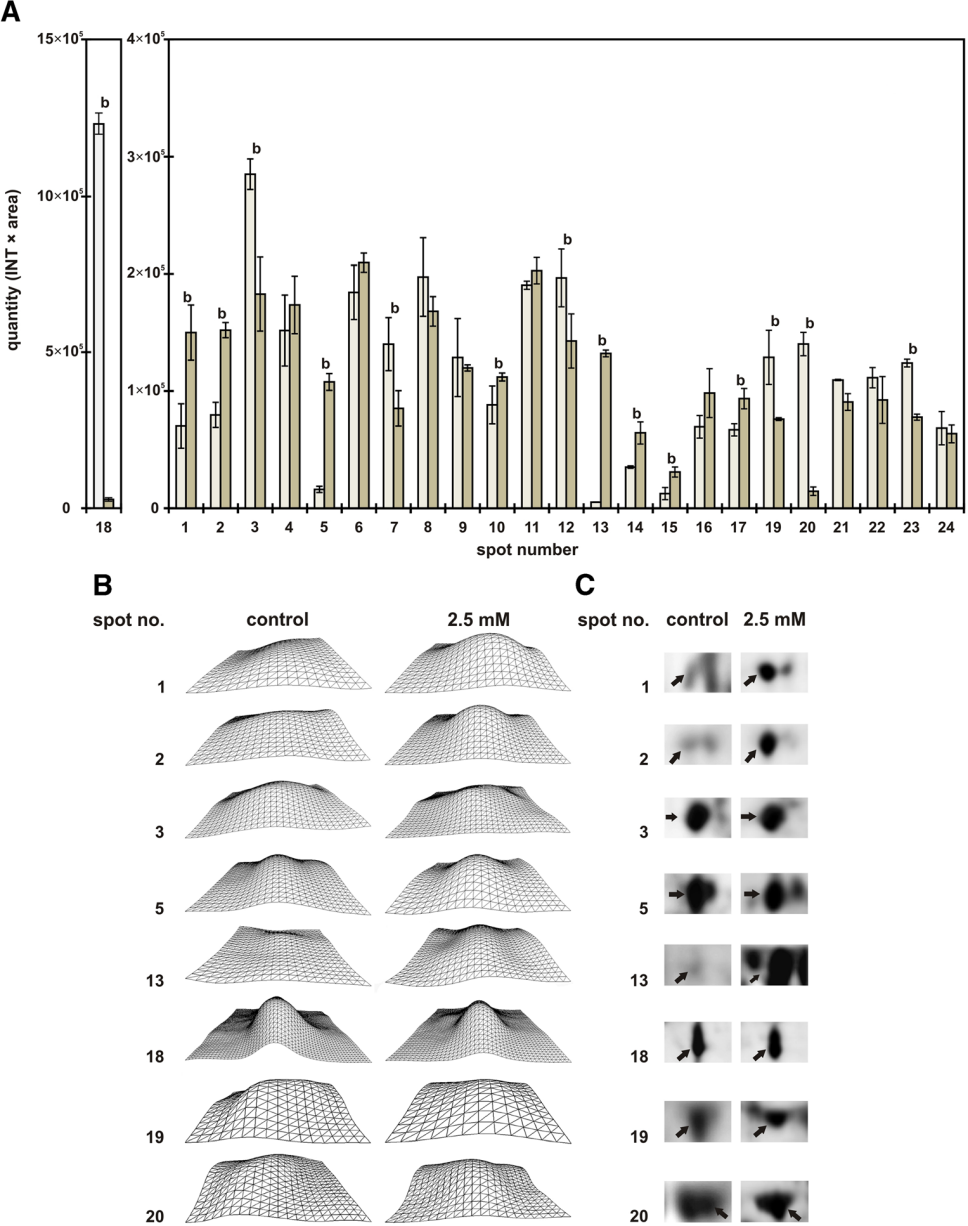
The average intensity of specific protein spots (PDQuest, Bio-Rad) for control roots (□) and levofloxacin (2.5 mM of soil)-treated roots (■). **a** Spot numbers (compare Table 3 ). **b** 3D profile of specific spots’ optical intensities: 1—full = protein LlR18A, 2—class III chitinase, 3—14-3-3-like protein A, 5—ascorbate peroxidase, 13—full = alcohol dehydrogenase 1, 18—glyceraldehyde-3-phosphate dehydrogenase, 19—lipoxygenase, 20—nucleotide-binding subunit of vacuolar ATPase. **c** Plain view of selected spots on 2D gels

The toxicity of LEV was determined in regard to protein expression in 12-day-old roots of yellow lupin. Identification of 24 proteins was carried out, which may be involved in developing yellow lupin resistance to LEV toxicity. The results are presented in Table 3, in which proteins were categorised into groups depending on their function. The following functional groups of proteins were identified: defence and response to stress, growth, energy and carbohydrate metabolism, detoxication, oxygen supply, signal transduction, translation control and protein pump. The largest group was that of proteins responsible for defence and plant reaction to environmental stress. The quantities of the most members of this group (five out of nine) increased as a result of levofloxacin treatment. Among the other members of this group, there were both those which decreased (nos. 3, 19) and those which remained constant (16, 6). Seven proteins (protein nos. 3, 7, 12, 18, 19, 20, 23) were found to show increased expression in control roots, and synthesis of eight proteins (nos. 1, 2, 5, 10, 13, 14, 15, 17) decreased (Fig. 6a). The expression of the remaining proteins (nos. 4, 6, 8, 9, 11, 16, 21, 22, 24) did not change (Fig. 6a–c; Table 3).

**Table 3:** Proteins identified by LC-MS-MS/MS analyses

Spot no.	Protein name	Species	Protein ID	Function	Mascot score
1	Full = protein LlR18A	*Lupinus luteus*	gi 1730080	Defence response	230
2	Class III chitinase	*Lupinus albus*	gi 2853142	Defence and stress responses	152
3	14-3-3-like protein A	*Glycine max*	gi 351720808	Metabolic regulation, stress responses	1126
4	Full = 60S acidic ribosomal protein P0	*Lupinus luteus*	gi 1710585	Translation control mechanisms, protein turnover	489
5	Ascorbate peroxidase	*Glycine max*	gi 4406539	Antioxidant, defence and stress responses	276
6	Germin-like protein 3	*Glycine max*	gi 196122010	Development and defence	121
7	Expansin-B3-like precursor	*Glycine max*	gi 387528019	Development	134
8	Stem 28 kDa glycoprotein precursor, putative	*Ricinus communis*	gi 255549796	Vegetative storage protein, acid phosphatase activity	123
9	Malate dehydrogenase 1, mitochondrial, partial	*Glycine soja*	gi 7343118888	Energy metabolism	363
10	Aspartate aminotransferase P1	*Lupinus angusitifolius*	gi 168324	Carbon and nitrogen metabolism	343
11	UDP-d-glucuronate carboxy-lyase	*Pisum sativum*	gi 13591616	Energy metabolism	232
12	Fructose-bisphosphate aldolase, cytoplasmic isozyme	*Glycine soja*	gi 734419305	Energy metabolism	905
13	Full = alcohol dehydrogenase 1	*Pisum sativum*	gi 113361	Protection against hypoxic metabolism	587
14	Enolase	*Lupinus luteus*	gi 6996529	Carbohydrate and energy metabolism, stress responses	1172
15	Leghemoglobin reductase-like	*Glycine max*	gi 356565179	Metabolism, provide oxygen	1042
16	Extensin peroxidase	*Lupinus albus*	gi 27448342	Development, stress responses	216
17	Heat shock cognate protein 80-like	*Glycine max*	gi 356552478	Stress responses, defence	2652
18	Glyceraldehyde-3-phosphate dehydrogenase	*Lupinus albus*	gi 62816190	Carbohydrate and energy metabolism	882
19	Lipoxygenase	*Pisum sativum*	gi 2459611	Metabolism, stress responses, defence, signal transduction	71
20	Nucleotide-binding subunit of vacuolar ATPase	*Arabidopsis thaliana*	gi 166627	Proton pomp, ions and metabolite transport	95
21	F1 ATPase	*Pisum sativum*	gi 2116558	Energy metabolism	113
22	Actin	*Oryza sativa*	gi 20329	Development, resistance, elongation and differentiation of the cell	107
23	Pyruvate dehydrogenase E1 component subunit alpha, mitochondrial-like	*Glycine max*	gi 356526868	Carbohydrate and energy metabolism	444
24	Peptidyl-tRNA hydrolase 2, mitochondrial	*Beta vulgaris*	gi 731320994	Transaction factor, apoptosis	175

## Discussion

Improvement of soil quality by fertilisation with manure is the main cause of contamination of agricultural land with antibiotics. VAs introduced to soil accumulate in it. They have been found in arable land in concentrations ranging from several milligrams to several grams per kilogram of soil under study (Du and Liu 2012). Plants grown in soil contaminated with antibiotics take them up and accumulate them in various organs. Hu et al. (2010) showed antibiotics to be present at the highest concentration in leaves, followed by shoots and roots. They also pointed out that growth and development of plants affects the distribution of antibiotics in tissues. VAs absorbed by plants may inhibit photosynthesis, growth of shoots and roots, reduce the leaf area and increase the production of ROS (Di Marco et al. 2014). EC_25_ and EC_50_ determined for the growth of roots of yellow lupin in this study were 0.2 and 0.67 mM (Table 1), respectively. Hillis et al. (2011) found EC_25_ and EC_50_ for the length of *Lactuca sativa*, *Medicago sativa* and *Daucus carota* roots to range from 39 μg L^−1^ (i.e. 1.08 × 10^−5^ mM) to over 10,000 μg L^−1^ (i.e. 0.00277 mM). The toxicity of LEV towards shoots was higher and ranged from 0.17 to over 1.24 mM for EC_25_ and EC_50_, respectively. Our study has also shown LEV to be more toxic towards roots than towards shoots (Tables 1 and 2). However, the LEV toxicity determined in this study was much lower than that reported by Hillis et al. (2011). This probably resulted from the differences in the way LEV was applied. We applied it directly to the reference soil in a PHYTOTOXKIT. It must be emphasised that soil can limit sorption of antibiotics, thereby reducing their reactivity and biological availability. Moreover, the content of organic carbon and clay in soil, the ionic strength and the pH can change the sorption mechanisms (Wegst-Uhrich et al. 2014). There are also strong sorptive interactions between drugs and soil, which may prevent antibiotic release and dispersion. These properties determine fate of the drugs in the environment and also limit antibiotic bioavailability (Hu et al. 2010). Moreover, absorption of antibiotics by plants is affected by the type of VAs, plant species and their root system. The uptake of pharmaceutics from the environment by roots and their biological activity *in planta* depends on the *n*-octanol-water partition coefficient of the VAs (Seo et al. 2010). The toxicity of LEV may be suggestive of poorly developed stress tolerance mechanisms in seedlings.

Antibiotics absorbed by plants induce oxidative stress (Di Marco et al. 2014) whose level can be determined by establishing the content of ROS (O_2_ **∙** ^**−**^, ∙OH, H_2_O_2_, ^1^O_2_) in plant cells. Reactions catalysed by oxidative stress enzymes create the main mechanism of scavenging and neutralising ROS (Wang et al. 2014). Adverse environmental factors may trigger an oxidative burst—an intracellular increase in the ROS level (Sofo et al. 2015). On the other hand, a change in the activity of the oxidative stress enzymes may indicate increased production of ROS in cells (Wen et al. 2012).

The activity of two oxidoreductases, guaiacol peroxidase (GPX) and catalase (CAT), are upregulated in lupin seedlings grown in soil contaminated with levofloxacin (Fig. 2), and the rate of this stimulation depends on antibiotic concentration in soil (Fig. 2a–f). There are no literature data on levofloxacin EC_50_ for GPX and CAT (Table 1). According to our findings, catalase is a better toxicity biomarker than peroxidase. However, it must be emphasised that the activity of GPX and CAT in 12-day-old roots of lupin increased nine and four times, respectively, compared to the activity of GPX and CAT in roots of control seedlings (Fig. 2). An increase in reductase activity in tissues indicates that the amount of ROS in levofloxacin affected cell increases (Sofo et al. 2015). A study carried out by Gujrathi and Linden (2005) revealed an association between oxidative stress and increased production of antioxidative enzymes in roots. Contamination of soil with tetracycline at low concentrations (from 0.5 to 10 mg L^−1^, i.e. from 1.1 × 10^−3^ to 2 × 10^−2^ mM or 25 to 300 mg L^−1^) results in a small increase in the activity of GPX and CAT in wheat roots. An increase in tetracycline concentration from 25 to 300 mg L^−1^(i.e. 6 × 10^−2^ to 0.7 mM) increases the activity of antioxidative enzymes by as much as 629.7 and 528.5% for CAT and GPX, respectively (Xie et al. 2011). A similar increase in the activity of the enzymes was observed in 12-day-old roots of lupin when soil was contaminated with a 2 mM (140 mg kg^−1^ of soil) solution of levofloxacin (GPX—663.1% and CAT—278.2%; Fig. 2). Di Marco et al. (2014) also reported an increase in the antioxidative enzyme activity in plants which grew in soil contaminated with antibiotics. The toxicity coefficients determined for catalase and peroxidase indicate that the toxicity units grow significantly when soil is contaminated with LEV at low concentrations (Tables 1 and 2). The metabolic responses of seedlings are much more rapid than the morphological changes. Under normal environmental conditions, the production and neutralisation of oxygen metabolites, potentially toxic, is balanced and the amounts of these molecules are strictly limited. At low levels, non-toxic to cells, reactive oxygen species can be used as signal molecules (Baxter et al. 2013). An increase in the ROS level may result in DNA, protein and lipid damage (Krishnamurthy and Rathinasabapathi 2013) and, ultimately, in programmed cell death (PCD) (Petrov et al. 2015). Visualisation (Fig. 3) of ROS demonstrated the toxicity of levofloxacin (2.5 mM) and the presence of oxidative stress in lupin roots. The oxidative burst is visible in cells as early as after 24 h of plant growth in soil contaminated with the antibiotic. Microscopic photographs show that ROS are first distributed fairly evenly in the cellular space (Fig. 3); however, after 2 days they are mainly localised along the cell wall. Non-specific metabolic disturbances are observed in root cells. The effect of drugs (diclofenac, paracetamol) on stress induction in cells has been demonstrated earlier by Kummerová et al. (2016). Reactive oxygen species and reactive nitrogen species (RNS) have been detected in stressed *Lemna minor*, especially in the tips and elongation zone of roots. Visualisation of reactive oxygen species in lupin roots 48 h after LEV was added to soil shows that they had accumulated near the cell membrane and cell wall (Fig. 3). ROS produced in plants under stress conditions cause direct or indirect oxidative modification of proteins (Das and Roychoudhury 2014).

Proteomic studies comparing plant proteins in extreme and normal conditions confirm the toxicity of levofloxacin. Depending on the tolerance of plants to abiotic stress, protective proteins can accumulate in them with different intensity (Rodziewicz et al. 2014). Changes of gene expression and protein accumulation are among the first defence reactions of plants to abiotic stress (Gong et al. 2015). This study shows the effect of levofloxacin on changes in the lupin root proteome (Figs. 4, 5 and 6). Among the 24 identified proteins (Table 3), there were nine directly related to the defence and response of plants to abiotic stress (Fig. 4a, b). The 2D maps show the accumulation of protective proteins and the ones related to stress responses; these were full = protein LlR18A, class III chitinase, ascorbate peroxidase, germin-like protein 3, extensin peroxidase and heat shock cognate protein 80-like (Table 3). The identified proteins serve roughly the same function, however, through different mechanisms. Chitinases are PR (pathogenesis-related) proteins whose role was initially identified only as plant protection against pathogens (viruses, fungi, bacteria) and pests (Grover 2012; Laribi-Habchi et al. 2015). Shrimp shell chitinase and chitin, as well as chitinase produced by *Bacillus licheniformis* (ChiA), have similar activity (Benhabiles et al. 2012, 2013a; Laribi-Habchi et al. 2015). Due to its antimicrobial properties, chitosan has been applied as a fruit preservative (Benhabiles et al. 2013b, 2013c). Moreover, chitin and its derivatives show cytotoxic action towards THP-1 (human monocytic leukaemia cell line), Hep2 (human larynx carcinoma cell line) and Rd cells (human embryo rhabdomyosarcoma cell line) (Salah et al. 2013; Bouhenna et al. 2015). Decrease in molecular mass of chitin decreases its affinity towards chitosan (Dziril et al. 2015) and enhances its anticancer activity (Salah et al. 2013). It has been shown that synthesis of chitinases is also induced under abiotic stress conditions, such as drought, salinity, the presence of heavy metals (Kasprzewska 2003) and osmotic stress (Grover 2012). Kadouche et al. (2012) have demonstrated that chitosan can facilitate the ecological removal of heavy metals. When applied in a suspension containing also hydroxyapatite and metals, chitosan acts as flocculant and accelerates the sedimentation process. The accumulation of class III chitinase (IF3) in roots of seedlings which grow in soil contaminated with LEV proves the involvement of a plant defence reaction (Fig. 6a–c). An increase in the expression of the class III chitinase gene was also shown in organs of *Arabidopsis thaliana*, which were exposed to environmental stresses, especially salt stress (Takenaka et al. 2009).The level of ROS increases in plants exposed to environmental stress and can cause cell damage. The glutathione-ascorbate cycle plays a crucial role in antioxidative defence mechanisms, in which ascorbate peroxidase catalyses transformation of H_2_O_2_ to H_2_O (Caverzan et al. 2012). Chai et al. (2011) observed an increase in the level of ascorbate peroxidase in *A. thaliana* exposed to abiotic stress*.* A similar accumulation of APX takes place in roots of lupin grown in soil contaminated with levofloxacin (Fig. 6; Table 3). A glycoprotein called germin-like protein 3 (GLP) is involved in the defence reaction against the toxic action of H_2_O_2_. The activity of GLPs is associated with enzymatic activity of OXO and superoxide dismutase (production of H_2_O_2_), ADP glucose pyrophosphatase/phosphodiesterase (AGPPase) and serine protease inhibitors (Wang et al. 2013). Cell wall proteins, such as extensin proteins, take part in plant response to various stressors (Lamport et al. 2011). Extensins can mediate a reaction of H_2_O_2_ binding by peroxidase (Brownleader et al. 2000). Increasing the content of H_2_O_2_ in cells induces the binding of extensins to peroxidases (Prxs—class III plant peroxidases), >which produce an insoluble network (EPs). EPs perform a defensive function by reinforcing the cell wall (Almagro et al. 2009). There is a molecular chaperon, heat shock protein 80-like (hsc80), which is responsible for maintaining the proper structure and regulation of specific target proteins. The hsc80 protein plays a key role in the regulation of the cell cycle and signal transduction. Moreover, it is indirectly responsible for maintaining cellular homeostasis during plant response to stress (Tran et al. 2015). Accumulation of the hsc80 protein in roots may indicate that some additional processes are triggered which are necessary to protect cells from the stressor.

Soil supplementation with levofloxacin reduces the accumulation of 14-3-3-like protein A in lupin roots. 14-3-3 Proteins play a role in a plant’s response to stress by regulating the action of many proteins involved in signal transduction (Roberts et al. 2002). However, regulation of nitrogen and carbon metabolism in plants is the main role of 14-3-3-like proteins (Diaz et al. 2011). Reduction of protein synthesis may be a sign of sensitivity and intolerance of lupin seedlings to soil contamination with antibiotics (Figs. 4, 5 and 6). A slight decrease in the accumulation of the 14-3-3 proteins was observed by Laino et al. (2010) and Hurkman et al. (2009) in wheat subjected to thermal stress. Synthesis of some proteins responsible for the regulation of energy metabolism was reduced. The activity of the glycolytic enzyme, glyceraldehyde-3-phosphate dehydrogenase, lipoxygenase, pyruvate dehydrogenase, leghemoglobin reductase-like and F1 ATPase was found to decrease. At the same time, studies revealed an accumulation of enolase, which is a protein responsible for carbohydrate metabolism. Enolase takes part in the process of glycolysis and catalyses the transformation of 2-phosphoglycerate (2PEP) into phosphoenolpyruvate (PEP) (dehydration) (Pan et al. 2013).

## Conclusion

The wide range of changes in protein synthesis in lupin roots indicate the diversity of plant metabolic reactions affected by soil contamination with antibiotics and suggest lupin is a plant with a rather high sensitivity to such stress. The contents and distribution of ROS suggest the antibiotic-induced impairment of redox reaction network, particularly in the vicinity of the cell membrane and cell wall. Guaiacol peroxidase and catalase are sensitive indicators of levofloxacin toxicity.

On a morphological level, the levofloxacin soil contamination may result in over 50% inhibition of root and shoot growth and a similar reduction in seedling fresh mass. On the other hand, the dry mass of levofloxacin-affected seedlings increases by over 50%.

The values of toxicity indexes and EC_50_ for levofloxacin induced disturbances in lupin are given.

## Abbreviation

CAT: Catalase
DCF: Dichlorofluorescein
FQ: Fluoroquinolone
GPX: Guaiacol peroxidase
LEV: Levofloxacin
ROS: Reactive oxygen species
TU: Toxicity indexes
VA: Veterinary antibiotics
